# Site selection optimization for 100% renewable energy sources

**DOI:** 10.1007/s11356-024-32733-z

**Published:** 2024-03-08

**Authors:** Onur Derse, Ebru Yilmaz

**Affiliations:** 1 Department of Industrial Engineering, Faculty of Engineering, Tarsus University, Tarsus, 33400 Mersin, Turkey; 2 Department of Industrial Engineering, Faculty of Engineering, Cukurova University, Sarıçam, 01250 Adana, Turkey

**Keywords:** Renewable energy, Site selection, Optimization, Goal programming model, RegARIMA method

## Abstract

The increase in the use of Renewable Energy Sources (RES) provides many advantages such as reducing the environmental problems and sustainability. In this study, a long-term optimum RES settlement strategic plan is conducted for 81 provinces in Turkey by considering real data. Biomass energy, solar energy, hydroelectric energy, geothermal energy, and wind energy are considered RES sources. Energy consumption until the 2050 year is estimated with the regARIMA method, and then a weighted goal programming model was developed in which the outputs of the regARIMA method and risk analysis are integrated. The results of the regARIMA method are tested, and the test results indicate that an *R*^2^ value close to 1 indicates that the model is suitable, a low and negative MPE value is neutral, and a MAPE value below 4% indicates high accuracy of the model. Using GAMS 23.5 optimization software program, the developed weighted goal programming model is solved optimally. In this integrated model developed, the objectives of minimizing the installation time, minimizing the investment cost, minimizing the annual cost, maximizing the carbon emission reduction, maximizing the usage time, and minimizing the risk are considered. When the results obtained regarding the number of installations according to the model are examined, the decisions are made for 53% wind energy, 23% biomass energy, 13% hydroelectric energy, 9% solar energy, and 2% geothermal energy. Computational results show that the effective solutions are obtained by minimizing the sum of goals values, covering all provinces in Turkey, and considering real data.

## Introduction

Benefits such as reducing environmental problems and ensuring sustainability are increasing the use of renewable energy sources. Renewable energy sources have evolved significantly over the last decades (Zhou et al. 2021), and the role of renewable energy sources tends to be increasing (Michalak and Dziugiewicz 2018). Renewable energy sources include biomass energy, wave energy, tidal energy, solar energy, hydroelectric energy, hydrogen energy, geothermal energy, wind energy, and hybrid energy systems consisting of the integration of these energies.

The use of renewable energy sources is becoming more widespread in Turkey. Turkey is one of the fastest-growing energy markets among OECD (Organisation for Economic Co-operation and Development) countries. Therefore, Turkey is an attractive center for energy companies and investors. However, the current energy production in Turkey is not sufficient for the available energy needs, so it is aimed to increase the ratio of renewable energy sources in the total energy production share (Erdin and Ozkaya 2019).

Deciding on problems such as facility location selection in renewable energy sources is one of the most important feasibility parameters for investment (Erdin and Ozkaya 2019). The site location problem is a strategic decision, and in order to make this decision correctly, the energy forecast for the next period must also be performed. Thus, renewable energy-based mathematical programming models covering energy forecasts should be handled.

Balancing energy demand and energy supply becomes increasingly difficult over time due to energy supply is uncertain (Weitzel and Glock 2018). Over the years, the electricity demand is constantly increasing, so it is necessary to establish a forecast model to understand future consumption (Jain et al. 2018). In recent years, the world’s energy consumption has increased rapidly due to main changes in industry and economy, and accurate demand estimates have become essential to develop an optimal strategy including the improvement of the economy (De Oliveira and Oliveira 2018). There are many different methods used to predict the energy demand correctly. Some of these methods are Autoregressive Integrated Moving Average (ARIMA), regression models, Artificial Neural Networks (ANN), and a regression model with ARIMA errors (regARIMA). The regARIMA method focuses on the special time series regression field, which is used to estimate a time series with the help of common variables containing items with a time series structure (Clark et al. 2020). Li et al. (2020) employ the regARIMA model using the values ​​of Root Mean Square Error (RMSE), Mean Absolute Error (MAE), and Mean Absolute Percent Error (MAPE). Miswan et al. (2016) use the regARIMA model, which has been proven in the context of many estimates, in estimating the electric load demand by considering the temperature-independent variable.

Generally, mathematical programming models are used in the problems considering renewable energy sources. Examining the different works from the related literature, Zamboni et al. (2009a), Zamboni et al. (2009b), Ren et al. (2016), Fattahi and Govindan (2018) for biomass energy, Moarefdoost et al. (2017) for wave energy, De La Torre and Conejo (2005) for tidal energy, Schwarz et al. (2018) for solar energy, Yu et al. (1998) and Siqueira et al. (2006) for hydroelectric energy, Han et al. (2012), Derse et al. (2022), and Güler et al. (2021) for hydrogen energy, Sigurdardottir et al. (2015) for geothermal energy, Jones and Wall (2016), Chen et al. (2018), and Derse and Yılmaz (2022) for wind energy, Kuznia et al. (2013) and Shi et al. (2017) for hybrid energy, addressed mathematical programming models.

The most commonly used quantitative method in dealing with RES problems is mathematical programming methods. Considering the mathematical programming models in the studies examined, it is seen that the most discussed purpose is the minimization of the total cost. The general issues in the studies reviewed can be summarized in Table 1.

**Table 1:** Objective function and constraints in studies on mathematical programming model

	Objective function	Constraints	Authors
Minimize	Total cost of the system	*Capacity constraints* *Constraints on feedstock fields* *Constraint on demand cities*	Huang et al. (2010)
		*Constraints for utility supply network* *Constraints for hydrogen supply network*	Hwangbo et al. (2016)
		*Mass-balance constraints* *Capacity constraints* *Constraints of utility supply network*	Hwangbo et al. (2017)
		*Capacity constraints* *Assignment constraints* *Budget constraints* *Reliability constraints*	Salimi and Vahdani (2018)
		*Capacity constraints* *Resource allocation constraints* *Demand constraints* *Personnel constraints*	Derse et al. (2022)
	Logistics and carbon cost	*Balancing constraints* *Capacity constraints*	Sadeghi and Haapala (2019)
	Environmental impact	*Mass balance constraints* *Energy balance constraints* *Economic analysis constraints* *Life cycle environmental impact constraints*	Gebreslassie et al. (2013)
	Transportation cost	*Technical constraints*	André et al. (2013)
Maximize	Profit	*Technical operating constraints*	De La Torre and Conejo (2005)
		*Physical constraints* *Operational constraints*	De Ladurantaye et al. (2009)
		*Mass balance constraints* *Energy balance constraints* *Economic analysis constraints* *Life cycle environmental impact constraints*	Gebreslassie et al. (2013)
		*Production capacity constraints* *Sustainability constraints* *Mass balance constraints* *Demand constraint*	Sigurdardottir et al. (2015)

Studies on increasing renewable energy capacities and studies on the concept of 100% renewable energy are emerging. The capacity expansion planning problem for the renewable energy industry includes decisions related to the optimal mix of different facility types, where each facility should be built, and capacity expansion decisions across the planning horizon for each facility (San Cristóbal 2012). The concept of 100% renewable energy is based on meeting all of the energy provided in the applied region with renewable energy sources. The 100% renewable energy system reduces the energy supply cost while reducing dependence on energy imports and carbon emissions (Kilickaplan et al. 2017). Connolly et al. (2011) address four 100% renewable energy scenarios based on 100% biomass energy, based on 100% hydrogen energy, maximizing the use of 100% renewable generated electricity, and based on the combination of each scenario. In Mathiesen et al. (2011), analyses and results of 100% renewable energy system design until 2050 year are presented. Two goals are determined with the 100% goal. To reach 67% in 2015 year and 85% in 2030 year is aimed, and analyses are carried out based on these goals. Ćosić et al. (2012) investigate two renewable energy scenarios in Macedonia where a 50% renewable energy system is aimed for 2030 year and a 100% renewable energy system is aimed for 2050 year. The results show that the renewable energy system based on 50% is more suitable. Chang (2015) proposes a goal programming model to deal with renewable energy’s capacity increase planning problem. The study aims to locate different types of facilities in proper locations in order to minimize total deviations from predefined goals, including power generated, investment cost, avoided emissions, jobs created, operating and maintenance costs, distance safety, and social acceptance. Papaefthymiou and Dragoon (2016) present an analysis for three different time intervals determined in their study. They examined the renewable energy system of 10% in the short term, 50% in the medium term, and 100% in the long term. Zografidou et al. (2016) develop a weighted goal programming model by considering various criteria. The results reveal that social and environmental criteria must be weighted more than economic ones to achieve maximum efficiency. Kilickaplan et al. (2017) analyze Turkey’s transition to 100% renewable energy by 2050 year using a model. The study aims to show how an optimum transit route can meet the electricity, water, and non-energy industrial gas demands with a 100% renewable energy system in the medium and long term by addressing the regions of Turkey. Hansen et al. (2019) discuss the problem of transitioning Germany’s energy system to 100% renewable energy towards 2050 year based on the decisions determined by Germany government to increase the share of renewable energy and to disable nuclear energy. Thellufsen et al. (2020) present a methodology for designing Smart Energy Cities in the context of 100% renewable energy at the national level. The study shows how the transition to a Smart Energy City is possible, fitting into the 100% renewable energy context of Denmark and Europe. Arévalo et al. (2022) study a technical–economic study of 100% renewable energy resources is carried out. Estimations are made for the years 2030 and 2050, taking into account the consumption data of 2011–2020. The study focuses on meeting the energy demand of the islands with renewable resource. Cheng et al. (2022) examine the future of renewable energy in Japan. This study shows that it has sufficient capacity to provide 100% renewable electricity. Provided that obstacles to mass deployment of solar photovoltaics and offshore wind are overcome, Japan could become self-sufficient in electricity supply. Lund et al. (2022) present a strategy to achieve a completely decarbonised Danish energy system by 2045. Østergaard et al. (2022) synthesize the EnergyPLAN applications used for 100% RES by analyzing its use in both bibliometric and case-geographical terms and reviewing the developments in the topics discussed and the results obtained using EnergyPLAN. In their study, Akpan and Olanrewaju (2023) introduce the methodological or evaluation mechanism suitable for current and future 100% renewable energy analysis. It then examines energy modeling tools to determine their applicability to 100% renewable energy analysis. Johannsen et al. (2023) examine the transition to 100% renewable energy. The study emphasizes that this situation may be possible before 2050, but for this to happen, all investments must be sustainable and grid electricity must be completely decarbonized starting from 2030. Marocco et al. (2023) study is to investigate hydrogen storage and batteries to achieve a 100% renewable energy system. As a result of the study, it is revealed that hydrogen-based solutions are important in cost-effectively ensuring energy self-sufficiency in 100% renewable energy systems. Rey-Costa et al. (2023) present the largest geographical and longest time series analysis on the sizing of solar and wind in the context of the Australian National Electricity Market. It examines the additional solar, wind, and battery capacity needed to meet 100% of demand between 2010 and 2020.

In this study, an optimum site selection problem for the gradual transition to 100% renewable energy sources in Turkey is addressed, and this study aims to make a significant contribution to the literature by bringing together integrated methods so that renewable energy resources can be used more. The study evaluates 81 provinces in Turkey and considers biomass energy, solar energy, hydroelectric energy, geothermal energy, and wind energy as renewable energy sources. The study is handled at a strategic decision level and aims to realize the optimum design of meeting the energy needs in the areas considered for a long-term location selection problem and to make long-term energy forecasting covering the period until 2050. The aim of the study is to integrate and use the regARIMA model and testing phase, goal programming, and risk analysis methods.

In the first part of the study, general explanations and previous studies on energy estimation, previous studies on facility location selection by considering renewable energy sources, and previous studies on 100% renewable energy sources are discussed. In the second part of the study, the definition of the problem is given. The materials and methods used in the study are presented in the third part. The results specific to the problem discussed in the fourth part, the discussion in the fifth part, and the conclusion in the last part are included.

## Problem definition

**Fig. 1:**
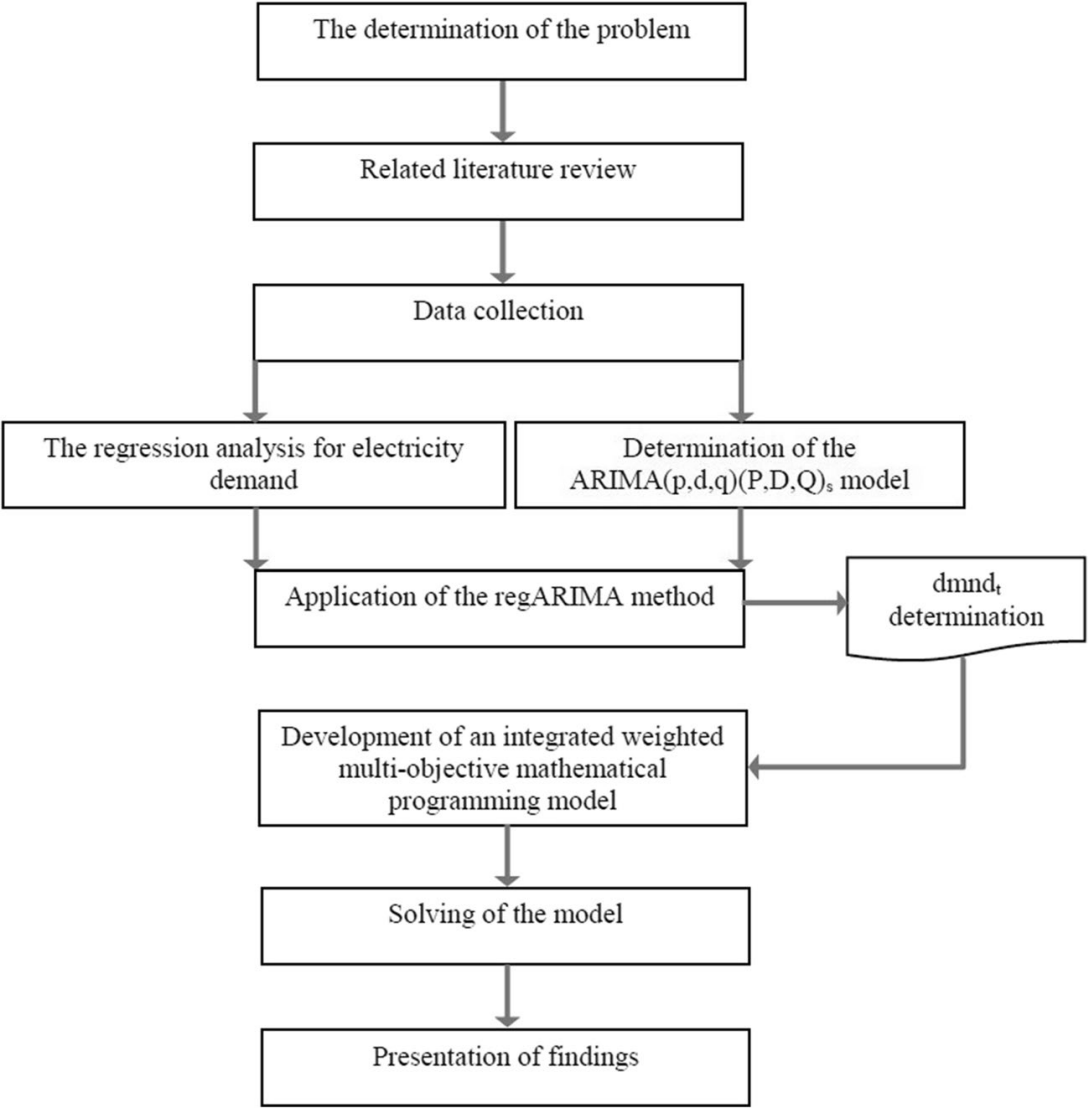
The flowchart of the article

In this study, an optimum site selection problem for the transition to 100% renewable energy sources is handled by evaluating all provinces in Turkey. Biomass energy, solar energy, hydroelectric energy, geothermal energy, and wind energy are discussed as renewable energy sources. The study is handled at a strategic decision level, and to make the optimum site selection of meeting the energy needs is planned for a long-term settlement problem. In this study, a weighted goal programming model is applied to make the optimum site selection. Turkey’s electricity demand is estimated by the regARIMA method until the 2050 year. The flow chart related to the developed system is shown in Fig. 1.

The study considers many real data and is based on the forecasting and site selection problem up to 2050. However, considering the limitations and assumptions of the study, hydroelectric energy data are assumed. The cost values used in the study are assumed. In addition, the goal values used in the study are assumed.

## Materials and methods

### RegARIMA method

RegARIMA models are one of the time series analysis methods in which the features of ARIMA and linear regression models are combined. To increase the accuracy of the model, the best ARIMA model is developed by combining with regression (Miswan et al. 2016).

In the ARIMA(p,d,q)(P,D,Q)_s_ model, which is a Seasonal Autoregressive Integrated Moving Average Model, parameters are shown in Table 2.

**Table 2:** Parameters of the ARIMA(p,d,q)(P,D,Q)_s_

Nomenclature	Description
*p*	autoregressive parameter
*q*	moving average
*d*	degree of difference
*P*	seasonal autoregressive parameter
*Q*	seasonal moving average
*D*	degree of seasonal variation
*s*	seasonal frequency
*a*	constant value
*y*_*t*_	dependent values
*X*_*t*_	independent values
*b*_*t*_	coefficients
*B*	backshift operator value
*e*	error term

While applying the regARIMA model, more than one independent variable is considered for a dependent variable and the JDemetra + package program is used for the regARIMA model developed in this study. As the dependent variable, Turkey’s monthly gross electricity consumption value between 2001 and 2020 years in kWh units until the end of August 2020 are considered. These data are obtained from “Turkish Electricity Transmission Corporation (TEİAŞ)”.

The literature is reviewed while deciding on the independent variables. Yumurtaci and Asmaz (2004) predict by considering the expansion of Turkey’s hydroelectric potential until the 2050 year. In this study, per person, electricity consumption growth rates are taken into account by considering population growth. Tunç et al (2006) state it is necessary to examine some economic indicators such as population, GDP (Gross Domestic Product), electricity consumption per person, or electricity consumption over GDP in order to analyze the energy sources, installed electrical power capacity, electrical energy production and consumption rates of Turkey, France, Germany, and Switzerland. Suganthi and Samuel (2012) address a large-scale literature review on energy demand forecasts in their study and stated that while using econometric models, parameters such as energy price, gross production, and population are generally related to energy demand. Kilickaplan et al. (2017) estimate what the electricity demand will be based on population.

By evaluating the studies in the literature, gross electricity production values, GDP, population, temperature, and electricity prices are taken as independent variables. The monthly gross electricity generation values ​​in Turkey are considered kWh units until the end of August 2020, as monthly values ​​between 2001 and 2020 years, and the data is obtained from “Turkish Electricity Transmission Corporation (TEİAŞ)”. GDP values ​​are considered in TL the currency unit of “Gross Domestic Product” from “Turkish Statistical Institute ((TURKSTAT), https://data.tuik.gov.tr/Bulten/Index?p=D%C3%B6nemsel-Gayrisafi-Yurt-%C4%B0%C3%A7i-Has%C4%B1la-III.-%C3%87eyrek:-Temmuz---Eyl%C3%BCl,-2020-33606&dil=1)” and the data is taken monthly and the monthly values ​​between 2001 and 2020 years are until the end of August 2020. Population values ​​are obtained from “Turkish Statistical Institute ((TURKSTAT), https://data.tuik.gov.tr/Kategori/GetKategori?p=nufus-ve-demografi-109&dil=1)” as the number of people from the total population in the “Population of Provinces by Years, 2000–2020”. The temperature values are obtained from “Turkish State Meteorological Service” taking into account the station name, year, month, and average temperature values (°C). The monthly average temperature values are calculated considering the monthly values between 2001 and 2020 years, until the end of August 2020 values. Electricity prices are obtained from electricity tariffs reported annually from “Turkish Electricity Distribution Corporation ((Türkiye Elektrik Dağıtım A.Ş., TEDAŞ), https://www.tedas.gov.tr/#!tedas_tarifeler)”. The values ​​between 2001 and 2005 years are taken into account from the values ​​at the beginning of the month by averaging the values ​​in the residential part of the uninominal tariff. The values ​​after 2006 year are considered in kr/kWh the currency unit from the values ​​in the unilateral tariff in the residential part of the consumers receiving energy from the distribution part until the end of August 2020 year.

### Mathematical programming model

In this study, the problem of optimum site selection of renewable energy sources for Turkey is discussed. A weighted multi-objective goal programming model is developed for this problem. For solving the developed mathematical programming model, GAMS 23.5 optimization software is used. Nomenclature related to the model is seen in Table 3.

**Table 3:** Nomenclature of the model

Nomenclature	Description
*I,S,T,J*	set of provinces, renewable energy sources, periods, goals
*pwr*_*si*_	current total installed power of the source
*ptn*_*si*_	total potential of the source
*dmnd*_*t*_	total demand amount calculated with the regARIMA method
*iygoal*	goal value of setup time
*ilgoal*	goal value of investment cost
*omgoal*	goal value of annual cost
*cogoal*	goal value of carbon emission reduction
*lfgoal*	goal value of usage time
*rskgoal*	goal value of risk
*pw*_*s*_	amount of power occurred when the source is established
*inv*_*st*_	installation cost
*lc*_*st*_	land cost
*oc*_*st*_	operation cost
*mc*_*st*_	maintenance cost
*co*_*s*_	carbon emission reduction amount when the source is established
*rsk*_*s*_	the risk level of the source calculated by risk analysis
*iy*_*s*_	setup time of source
*lf*_*s*_	usage time of source
*w*_*j*_	weight of goal value
*x*_*sit*_	integer decision variable showing how many power plants will be installed
*k*_*sit*_	decision variable showing the production power created by the established source
*cml*_*sit*_	total amount of power produced so far
*krln*_*t*_	decision variable showing the happened productive power up to time t
*sp*_*st*_	integer decision variable showing the total number of power plants opened from s source
${d}_{j}^{+}$	positive deviation value from goal j
${d}_{j}^{-}$	negative deviation value from goal j

1 $$Minimize({{\mathrm{w}}}_{1}{{\mathrm{d}}}_{1}^{+}+{{\mathrm{w}}}_{2}{{\mathrm{d}}}_{2}^{+}+{{\mathrm{w}}}_{3}{{\mathrm{d}}}_{3}^{+}+{{\mathrm{w}}}_{4}{{\mathrm{d}}}_{4}^{-}+{{\mathrm{w}}}_{5}{{\mathrm{d}}}_{5}^{-}+{{\mathrm{w}}}_{6}{{\mathrm{d}}}_{6}^{+} )$$

As the multi-objective goal programming model, minimization of the totals of setup time goal, investment cost goal, annual cost goal, carbon emission reduction goal, usage time goal, and risk goal are discussed, and in the objective in the Equation function (1), these items are weightily expressed respectively.

2 $$\begin{array}{cc}{{{\mathrm{x}}}_{{\mathrm{sit}}}}^{*}{{\mathrm{pw}}}_{{\mathrm{s}}}={{\mathrm{k}}}_{{\mathrm{sit}}}& ,\forall {\mathrm{s}},{\mathrm{i}},{\mathrm{t}}\end{array}$$

3 $$\begin{array}{cc}{\mathrm{k}}{\mathrm{s}}{\mathrm{i}}{\mathrm{t}} \, \le {{\mathrm{ptn}}}_{{\mathrm{si}}}-{{\mathrm{pwr}}}_{{\mathrm{si}}}& \mathrm{,t=1, }\forall {\mathrm{s}}\mathrm{,i}\end{array}$$

4 $$\begin{array}{cc}{{\mathrm{k}}}_{{{\mathrm{si}}}^{\mathrm{{\prime}}}{\mathrm{t}}+{1}^{\mathrm{{\prime}}}}\le \, {{\mathrm{ptn}}}_{{\mathrm{si}}}- \, {{\mathrm{pwr}}}_{{\mathrm{si}}}-{{\mathrm{cml}}}_{{\mathrm{sit}}}& \mathrm{,}\forall {\mathrm{s}}\mathrm{,i,t}\end{array}$$

5 $$\begin{array}{cc}\sum\limits_{{\mathrm{t}}}{{\mathrm{k}}}_{{{\mathrm{si}}}^{\mathrm{{\prime}}}{\mathrm{t}}-{1}^{\mathrm{{\prime}}}}={{\mathrm{cml}}}_{{\mathrm{sit}}}& \mathrm{,}\mathrm{t=2,..., T, }\forall \mathrm{s,i}\end{array}$$

6 $$\begin{array}{cc}\sum\limits_{\mathrm{s}}\sum\limits_{{\mathrm{i}}}\sum\limits_{{\mathrm{t}}}{{\mathrm{k}}}_{{\mathrm{sit}}}= \mathrm{ } {{\mathrm{krln}}}_{{\mathrm{t}}}& \, \mathrm{,}\forall {\mathrm{t}}\end{array}$$

7 $$\begin{array}{cc}\sum\limits_{\mathrm{s}}\sum\limits_{{\mathrm{i}}}{\mathrm{pwr}}{\mathrm{s}}{\mathrm{i}}+{{\mathrm{krln}}}_{{\mathrm{t}}}\ge {{\mathrm{dmnd}}}_{{\mathrm{t}}}& \mathrm{,}\forall {\mathrm{t}}\end{array}$$

8 $$\sum_{\mathrm{s}}\sum_{{\mathrm{i}}}\sum_{{\mathrm{t}}}{{\mathrm{x}}}_{{\mathrm{sit}}} \, *{\mathrm{iy}}{\mathrm{s}}-{{\mathrm{d}}}_{1}^{+}+ {{\mathrm{d}}}_{1}^{-} \mathrm{ = }{\mathrm{iygoal}}$$

9 $$\sum_{\mathrm{s}}\sum_{{\mathrm{i}}}\sum_{{\mathrm{t}}}{{\mathrm{x}}}_{{\mathrm{sit}}}*{{\mathrm{inv}}}_{{\mathrm{s}}}\mathrm{+}\sum_{\mathrm{s}}\sum_{{\mathrm{i}}}\sum_{{\mathrm{t}}}{{\mathrm{x}}}_{{\mathrm{sit}}}*{1{\mathrm{c}}}_{{\mathrm{s}}}-{{\mathrm{d}}}_{2}^{+}\mathrm{+}{{\mathrm{d}}}_{2}^{-} \, = \mathrm{ } {\mathrm{ilgoal}}$$

10 $$\sum_{\mathrm{s}}\sum_{{\mathrm{i}}}\sum_{{\mathrm{t}}}{{\mathrm{x}}}_{{\mathrm{sit}}}*{{\mathrm{oc}}}_{{\mathrm{s}}}+ \sum_{\mathrm{s}}\sum_{{\mathrm{i}}}\sum_{{\mathrm{t}}}{{\mathrm{x}}}_{{\mathrm{sit}}}*{{\mathrm{mc}}}_{{\mathrm{s}}}-{{\mathrm{d}}}_{3}^{+}+ {{\mathrm{d}}}_{3}^{-}={\mathrm{omgoal}}$$

11 $$\sum_{\mathrm{s}}\sum_{{\mathrm{i}}}\sum_{{\mathrm{t}}}{{\mathrm{x}}}_{{\mathrm{sit}}}*{{\mathrm{co}}}_{{\mathrm{s}}}-{{\mathrm{d}}}_{4}^{+}+ {{\mathrm{d}}}_{4}^{-}={\mathrm{cogoal}}$$

12 $$\sum_{\mathrm{s}}\sum_{{\mathrm{i}}}\sum_{{\mathrm{t}}}{{\mathrm{x}}}_{{\mathrm{sit}}}*{1{\mathrm{f}}}_{{\mathrm{s}}}-{{\mathrm{d}}}_{5}^{+}+ {{\mathrm{d}}}_{5}^{-}={\mathrm{lfgoal}}$$

13 $$\sum_{\mathrm{s}}\sum_{{\mathrm{i}}}\sum_{{\mathrm{t}}}{{\mathrm{x}}}_{{\mathrm{sit}}} \, *{{\mathrm{rsk}}}_{{\mathrm{s}}}-{{\mathrm{d}}}_{6}^{+}+ {{\mathrm{d}}}_{6}^{-} \, ={\mathrm{rskgoal}}$$

14 $$\begin{array}{cc}\sum\limits_{\mathrm{i}}{{\mathrm{x}}}_{{\mathrm{sit}}}={{\mathrm{sp}}}_{{\mathrm{st}}}& \mathrm{,}\forall \mathrm{s,t}\end{array}$$

15 $$\sum_{\mathrm{j}}{{\mathrm{w}}}_{{\mathrm{j}}}= \mathrm{ 1}$$

16 $$\begin{array}{cc}{{\mathrm{x}}}_{{\mathrm{sit}}}\mathrm{, }{{\mathrm{sp}}}_{{\mathrm{st}}}\ge {0}\mathrm{ and integer}& \mathrm{,}\forall {\mathrm{s}}\mathrm{,i,t}\end{array}$$

17 $$\begin{array}{cc}{\mathrm{k}}{\mathrm{s}}{\mathrm{i}}{\mathrm{t}}\mathrm{, }{{\mathrm{cml}}}_{{\mathrm{sit}}}\mathrm{, }{{\mathrm{krln}}}_{{\mathrm{t}}}\mathrm{, }{{\mathrm{d}}}_{{\mathrm{j}}}^{+}\mathrm{, }{{\mathrm{d}}}_{{\mathrm{j}}}^{-}\ge {0}& \mathrm{,}\forall \mathrm{s,i,t}\mathrm{,j}\end{array}$$

Constraint (2) shows the power to be provided by the power plants that are decided to be established. Constraint (3) and Constraint (4) show that the power plants that are installed and planned to be established cannot exceed the provincial potential. Constraint (5) shows the cumulative sum of the installations to the current period on the basis of period and sources, and Constraint (6) shows the generation power of the total installed power plants up to that period. Constraint (7) shows that demand must be met. Constraint (8) shows the setup time goal, Constraint (9) and Constraint (10) show the cost goals, Constraint (11) shows the carbon reduction goal, Constraint (12) shows the usage time goal, and Constraint (13) shows the risk goal. Constraint (14) shows the total number of power plants installed on the basis of source and period. Constraint (15) shows that the sum of the weights cannot exceed 1. Constraint (16) defines positive and integer decision variables. Constraint (17) defines positive decision variables.

### Input of mathematical programming model

In this study, the index i consists of 81 provinces in Turkey. The index s refers to the renewable energy source index and consists of biomass energy, solar energy, hydroelectric energy, geothermal energy, and wind energy. The index t indicates the time and covers the years between 2021 and 2050 years. The j index symbolizes the goal index, and in this study, there are six goals including installation time, investment cost, annual cost, carbon emission reduction, usage time, and risk minimization.

Some parameter values are assumed in the developed mathematical programming models. These assumed values are presented in this section. One of the goal parameter values, iygoal, indicates the setup time goal for all power plants. It is entered as 500 because the value is intended to be at the minimum level. ilgoal is used as the investment cost goal, and the goal value is assumed to be 25 × 10^6^ currency units. omgoal is indicated as an annual cost goal, and the goal value is assumed to be 25 × 10^5^ currency units. cogoal expresses the goal of carbon emission reduction, and it is aimed to reduce 2 × 10^6^ tons of carbon emissions. lfgoal indicates the usage time goal, and the goal value is assumed to be 10,000 since the value is intended to be maximum. rskgoal shows the risk goal, and this value is entered as 0 since it is intended to be at the minimum level. The coefficients for the determined goals are expressed as w_j_. This weight is handled as user input. The weight coefficients considered are assumed to be 1 in total, respectively, as follows. Setup time goal weight value is 0.09, investment cost goal weight value is 0.26, annual cost goal weight value is 0.15, carbon emission reduction goal weight value is 0.19, usage time goal weight value is 0.11, and risk minimization goal weight value is 0.2.

In Table 4, pw_s_ shows the amount of power that the source can provide, co_s_ shows the amount of carbon emission reduction when the source is installed, lf_s_ shows the usage time values of the source, iy_s_ shows the annual installation time and the values are assumed, and rsk_s_ shows the values related to the risk level of the source and is obtained by 5 × 5 risk analysis method.

**Table 4:** Parameters based on renewable energy sources

Renewable energy sources by electricity production (s)/parameters	pw_s_ (MW)	co_s_ (TonCarbon/year)	lf_s_ (year)	iy_s_ (year)	rsk_s_
Biomass energy (BE)	21	4500	30	0.8	6
Solar energy (SE)	25	450	20	0.85	4
Hydroelectricity energy (HE)	35	500	25	2.5	9
Geothermal energy (GE)	20	3000	25	0.7	6
Wind energy (WE)	17	2000	30	0.5	4

In Table 5, inv_st_ is the source’s setup cost, lc_st_ is the source’s land cost, oc_st_ is the source’s operating cost, mc_st_ is the source’s maintenance cost, and these values are assumed.

**Table 5:** Cost parameters based on renewable energy sources

	inv_st_*10^2^ (currency unit)					lc_st_*10^2^ (currency unit)				
t/s	BE	SE	HE	GE	WE	BE	SE	HE	GE	WE
1	150	180	170	150	135	160	175	190	150	105
2	152.5	177.5	172	151	140	162	180	191	152.5	107
3	155	175	174	152	145	164	185	192.5	153	109
4	157.5	172.5	176	153	142.5	166	190	193	153.5	111
5	160	170	178	154	142.75	168	195	193.5	154	113
6	170	167.5	180	155	143	170	200	192.75	154.5	115
7	175	165	182	156	142.9	172	190	192	155	117
8	180	162.5	184	157	142.8	174	180	191.25	154	119
9	185	160	180	156.5	142.7	176	175	190.5	153	121
10	190	157.5	181.5	156	142.6	178	170	189.75	152	123
11	180	160	183	155.5	142.5	180	165	189	151	125
12	170	162.5	184.5	155	142.4	181.5	160	188.25	150	127
13	160	167.5	186	154.5	142.3	183	155	187.5	149	129
14	150	172.5	187.5	154	140.5	184.5	150	186.75	148	131
15	151.5	177.5	189	153.5	138.7	186	145	186	147	133
16	153	175	187.5	153	136.9	184	140	185.25	146	135
17	154.5	172.5	186	152.5	135.1	182	135	184.5	145	137
18	156	170	184.5	152	133.3	180	130	180	143.5	139
19	157.5	167.5	183	151.5	131.5	178	140	175.5	142	141
20	150	165	181.5	151	129.7	176	142.5	171	140.5	143
21	144.5	162.5	180	150.5	127.9	174	145	175	139	145
22	139	160	178.5	150	126.1	170	147.5	179	137.5	147
23	133.5	157.5	177	149.5	124.3	166	150	183	136	149
24	128	159.5	175.5	149	122.5	162	152.5	187	134.5	151
25	130	161.5	174	148.5	120.7	158	155	191	133	153
26	132	163.5	172.5	148	118.9	154	157.5	190	131.5	150
27	134	165.5	171	147.5	117.1	150	160	189	130	147
28	136	167.5	169.5	147	115.3	146	162.5	188	128.5	144
29	138	169.5	168	146.5	113.5	142	165	187	127	141
30	140	171.5	166.5	146	111.7	138	167.5	186	125.5	138

	oc_st_*10 (currency unit)					mc_st_*10 (currency unit)				
t/s	BE	SE	HE	GE	WE	BE	SE	HE	GE	WE
1	400	100	500	200	150	50	80	95	45	30
2	405	105	505	205	155	55	85	100	50	31.5
3	410	110	510	210	160	60	90	105	55	33
4	415	115	515	215	165	65	95	110	50	34.5
5	420	120	520	220	167.5	70	100	115	52.5	36
6	425	125	525	225	170	69	105	120	53	37.5
7	430	130	500	230	169	68	110	115	55	39
8	435	135	475	235	165	67	115	110	57	38.5
9	440	140	450	240	161	66	120	105	59	38
10	445	145	425	245	157	65	125	100	61	37.5
11	450	150	400	250	153	64	130	95	63	37
12	455	155	375	255	149	63	135	90	65	36.5
13	460	160	385	260	145	62	137.5	85	67	36
14	465	165	395	265	141	61	135	80	65	35.5
15	470	170	405	270	137	59.5	133	75	63	35
16	475	175	415	275	133	58	131	70	61	34.5
17	480	180	400	280	135	56.5	129	65	59	34
18	485	185	385	285	137	55	127	60	57	35
19	490	190	370	290	139	53.5	130	55	55	36
20	495	195	355	295	141	52	133	57.5	53	37
21	500	200	340	300	143	50.5	136	60	51	38
22	505	205	325	305	145	49	139	62.5	49	39
23	510	210	310	310	147	47.5	142	65	47	40
24	515	215	295	315	149	46	145	67.5	45	41
25	520	220	280	320	151	44.5	148	70	43	42
26	525	225	265	325	153	46	151	72.5	41	43
27	530	230	290	330	155	47.5	154	75	39	44
28	535	235	315	335	157	49	157	77.5	37	45
29	540	240	340	340	159	50.5	160	80	35	46
30	545	245	365	345	161	52	163	82.5	33	47

Currently, according to “Turkish Electricity Transmission Corporation ((TEİAŞ), https://www.teias.gov.tr/)” data, the share of renewable energy sources in daily production is approximately 26%. As in the studies of Mathiesen et al. (2011), Ćosić et al. (2012), Kilickaplan et al. (2017), and Hansen et al. (2019), in this study, to meet the electricity consumption by 100% renewable energy sources in 2050 year is aimed. In this context, dmnd_t_ data is entered into the system. dmnd(t) shows the expected total demand at time t, and the values calculated with the regARIMA are used. The values calculated on a monthly basis are added and are entered into the system annually. While the values are entered into the system, the goal for 2023 year is determined as 30%, and for 2035 year as 60%, and it is aimed to meet the demand at these rates. In 2050 year, the coefficients have been entered into the system so that 100% will be met.

pwr_si_ expresses the total installed power of the s source in the province i. As a result of the values ​​collected on the basis of provinces in Turkey, the total installed power for sources is obtained from “Turkish Electricity Transmission Corporation (TEİAŞ)”.

ptn_si_ shows the total potential of the s source in the province i. As a result of the values ​​collected on the basis of provinces in Turkey, the total potentials for the sources are handled. Biomass energy potential data obtained from “Republic of Turkey Ministry of Energy and Natural Resources (https://bepa.enerji.gov.tr/)” including the energy equivalent of animal wastes (TEP/year) data, the energy equivalent of municipal wastes (TEP/year) data, and the energy equivalent of herbal wastes (TEP/year) data is collected to form the energy equivalent (TEP/year) data of total wastes for each province in Turkey. Solar energy data obtained from the “Republic of Turkey Ministry of Energy and Natural Resources” is considered the solar energy potential in kWh/year for all provinces in Turkey. Hydroelectric energy data is assumed to be the same with solar data. The data from source potential values of provinces related to geothermal source potential published in Akkuş and Alan (2016) is used. Wind energy data obtained from the “Republic of Turkey Ministry of Energy and Natural Resources” is considered the MW values of the wind energy potential for all provinces in Turkey.

## Results

### Results for the RegARIMA method

In this study, more than one independent variable is considered for a dependent variable usingRegARIMA model. As the dependent variable, Turkey’s monthly gross electricity consumption values between 2001 and 2020 years are considered monthly values ​​in kWh units until the end of August 2020. Gross electricity production values, GDP, population, temperature, and electricity prices are used as independent variables.

While applying the RegARIMA model, 236 data are collected monthly from the beginning of 2001 year to the end of August 2020. The model is tested by dividing this data for training and testing. The model equation is created with approximately 84% data, and 37 months of data between August 2017 and August 2020 are tested. Since there is seasonality in the gross electricity consumption data while applying the ARIMA model, X13 analysis is performed in the JDemetra + package program.

In this study, it is seen that the model performed with JDemetra + and obtained according to the X13 analysis is ARIMA(1,1,1)(0,1,1)_12_.

The initial values of the established regression equation are shown in Table 6. According to the table, the temperature value is not statistically significant for the regression equation. For this reason, the temperature value is removed from the regression equation and reanalysis is applied.

**Table 6:** Regression values of the independent variables

Variables	Coefficient value	*p* value
Constant value	6,738,053,664	0.000
Gross electricity production value	1.00038	0.000
GDP	0.0006231	0.018
Population	− 109.81	0.000
Temperature	− 1,790,532	0.272
Electricity price	59,131,127	0.000

When the values obtained after removing the temperature value are examined, it is seen that the gross electricity production, GDP, population, and electricity prices are statistically significant for the equation, and these values are shown in Table 7.

**Table 7:** Regression values of the independent variables that are statistically significant

Variables	Coefficient value	*p* value
Constant value	6,469,213,262	0.000
Gross electricity production value	0.99884	0.000
GDP	0.0005544	0.030
Population	− 105.82	0.000
Electricity price	59,238,072	0.000

RegARIMA equation is obtained with regression analysis and ARIMA(1,1,1)(0,1,1)_12_ model. The generated equation is shown as Y = a + b_1_.X_1_ + b_2_.X_2_ + b_3_.X_3_ + b_4_.X_4_ + e. Y value is the monthly gross electricity consumption value which is the dependent variable. X_1_, X_2_, X_3_, and X_4_ values symbolize monthly gross electricity production, GDP, population, and electricity price, respectively. The b_1_, b_2_, b_3_, and b_4_ values specify the coefficients of monthly gross electricity production, GDP, population, and electricity prices, respectively. The value e defines the error term. The regARIMA equation obtained is shown in Eq. (18). Equation (19) shows the equation of the error term e; the value of B defines the backshift operator value.

18 $${\mathrm{Y}}=6469213262+0.99884.{{\mathrm{X}}}_{1}+0.0005544.{{\mathrm{X}}}_{2}-105.82.{{\mathrm{X}}}_{3}+59238072.{{\mathrm{X}}}_{3}+{\mathrm{e}}$$

19 $${\mathrm{e}}=\left(\left(1+0.672899.{\mathrm{B}}\right).\left(1+0.637239.{{\mathrm{B}}}^{12}\right)\right)/\left(1-0.170439.{\mathrm{B}}\right).\left(1-{\mathrm{B}}\right)$$

**Fig. 2:**
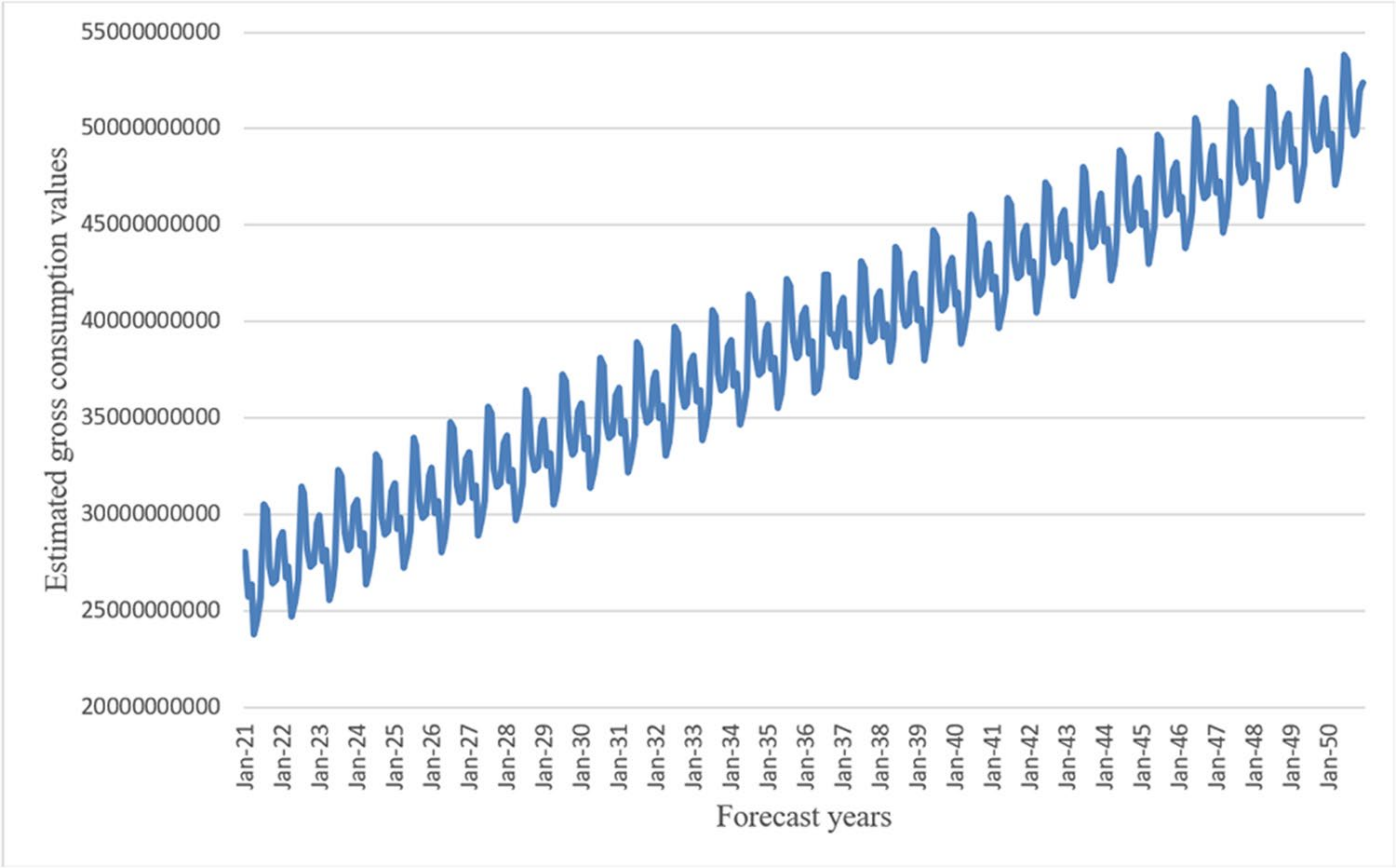
Gross electricity forecast values (kWh/year) until the 2050 year with the RegARIMA model

With the made RegARIMA model, the estimation is made until the 2050 year, and the result of the estimation values is obtained as in Fig. 2.

#### Test of the RegARIMA method’s results

When the R^2^, MPE, and MAPE values are examined as evaluation criteria for the RegARIMA model, the results are given in Table 8. R^2^ value close to 1 indicates that the model is suitable, low and negative MPE value is neutral, and MAPE value below 4% indicates high accuracy of the model.

**Table 8:** Some measurements for the model

Measurement	R^2^	MPE	MAPE
Value	0.94157756	− 3.866394086239	3.866394086239

### Results for risk analysis

Risk analysis is applied to be used in the developed mathematical programming model, and the 5 × 5 method is used for risk analysis. The 5 × 5 method is based on multiplying the probability of an event with its severity and determining the risk levels. A general risk table including assumed values for renewable energy sources discussed in Table 9 is made.

**Table 9:** Risk score for renewable energy sources

Renewable energy sources	Probability	Severity	Risk score
Biomass energy (BE)	2	3	6
Solar energy (SE)	2	2	4
Hydroelectricity energy (HE)	3	3	9
Geothermal energy (GE)	2	3	6
Wind energy (WE)	2	2	4

### Results for mathematical programming model

For the developed mathematical programming model, using the GAMS 23.5 optimization program, the optimum result is reached in 0.25 s using a computer with Intel(R) Core(TM) i5-10210U CPU @ 1.60 GHz 2.11 GHz processor. The model is formulated as weighted goal programming. The determined goals in the model are installation time minimization, investment cost minimization, annual cost minimization, carbon reduction amount maximization, usage time maximization, and risk minimization, respectively.

The x_sit_ obtained in the weight goal programming model is the decision variable that shows how many power plants will be established, k_sit_ shows the power generated by the installed sources, and sp_st_ defines the total number of power plants opened. Table 10 shows the results of the decision variables x_sit_, k_sit_, and sp_st_. At times not specified in this table, the results of the relevant decision variables appear as 0.

**Table 10:** Results of decision variables (x_sit_, k_sit_, and sp_st_)

		x_sit_								
	i / t	1	2	3	4	18	24	25	26	30
BE	1						3			
	53		99							
	69	100								
	sp_st_	100	99				3			
SE	29					23				
	43					55				
	sp_st_					78				
HE	52								26	
	62								27	
	73							21		
	77								38	
	sp_st_							21	91	
GE	3	17								
	63	2								
	65	1								
	sp_st_	20								
WE	1			52						
	15				3					
	17									66
	19				9					
	24	18								4
	33			48						
	42	82	13							
	43				11					
	57		87							
	60				74					
	74				3					
	sp_st_	100	100	100	100					70

	k_sit_									
	1	2	3	4	18	24	25	26	30	
BE						63				
		2079								
	2100									
SE					575					
					1375					
HE								910		
								945		
							735			
								1330		
GE	340									
	40									
	20									
WE			884							
				51						
									1122	
				153						
	306								68	
			816							
	1394	221								
				187						
		1479								
				1258						
				51						

**Fig. 3:**
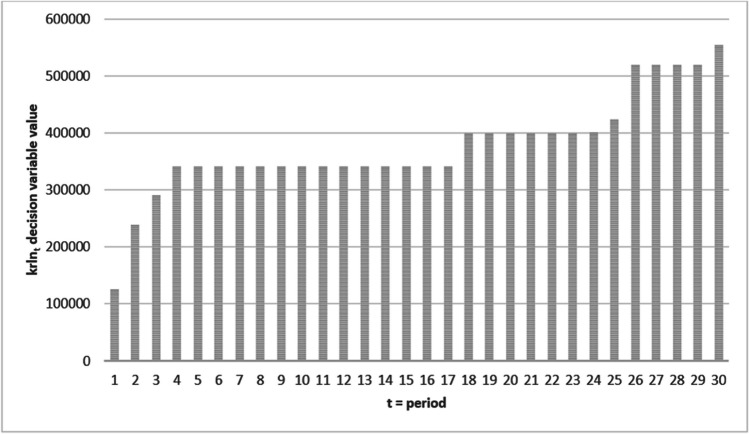
krln_t_ decision variable results

The decision variable krln_t_, which shows the cumulative sum between 2021 and 2050 years, is demonstrated in Fig. 3.

The goal values and deviations for the developed mathematical programming model are specified in Table 11. The goals determined in the developed weighted goal programming model are installation time minimization, investment cost minimization, annual cost minimization, carbon reduction amount maximization, usage time maximization, and risk minimization, respectively. According to the table, the objectives of minimization of investment cost, minimization of annual cost, maximization of carbon reduction amount, and maximization of usage time have been achieved, while the objectives of installation time minimization and risk minimization have not been achieved.

**Table 11:** The values of the goals and the deviations

	j = 1	j = 2	j = 3	j = 4	j = 5	j = 6
Values of goals	500	25*10^6^	25*10^5^	2*10^6^	10,000	0
Positive deviation values	256.9	0	0	100	15,020	4532
Negative deviation values	0	13*10^2^	185	0	0	0

## Discussion

In the study, the long-term planning is made for the transition to 100% renewable energy sources by using the integrated mathematical programming model with estimation while the site selection decision problem is handled.

In this study, a strategic plan is created for the transition to 100% renewable energy sources by 2050, and the study is implemented for Turkey. As a result of the study, it is seen that this strategy is applicable. As can be seen in the studies, it is thought that long-term appropriate strategies can be formed by carrying out similar studies in different countries.

In this study, the regARIMA method was used as the estimation method. The RegARIMA method is a more up-to-date approach compared to different estimation techniques. When the literature is examined, although mathematical programming models integrated with estimation are rare, an integrated mathematical programming model with regARIMA was not encountered.

In the developed goal programming model, investment cost, annual cost, amount of carbon reduction, usage time, installation time, and risk are considered goals in the study. When the studies for 100% RES are examined by considering the goal programming model; similarly, investment cost, operation and maintenance costs, and carbon emissions are considered in the studies by San Cristóbal (2012) and Chang (2015).

According to the results, the objectives of minimization of investment cost, minimization of annual cost, maximization of carbon reduction amount, and maximization of usage time have been achieved, while the objectives of installation time minimization and risk minimization have not been achieved. It can be seen that four objectives are met and two objectives are not achieved. The installation time minimization goal is very close, but there is a slight deviation from the value. The risk minimization goal value is entered into the system as 0. The reason for this is to challenge the models so that they can provide better risk results. The deviation from this goal is an expected result since the value 0 will be exceeded even when any installation decision is made. For this reason, the deviation of the risk goal occurs in the developed model.

## Conclusion

The importance and use of renewable energy sources are increasing day by day, and therefore, strategic plans and a long-term roadmap should be formed to take the right steps regarding renewable energy sources.

Turkey has a potential in terms of renewable energy sources, and a strategic plan is required to use these sources correctly. In this study, optimum settlement decisions for renewable energy sources including biomass energy, solar energy, hydroelectric energy, geothermal energy, and wind energy until the 2050 year are examined by considering 81 provinces in Turkey. In the study, many factors and methods are considered while making the optimum settlement decision. First of all, the regARIMA model is developed in the X13 analysis of the JDemetra + package program for energy consumption prediction until the 2050 year. In the developed RegARIMA model, as the dependent variable, Turkey’s monthly gross electricity consumption values ​​between 2001 and 2020 years are considered monthly values ​​in kWh units until the end of August 2020. The gross electricity production values, GDP, population, temperature, and electricity prices have been decided as an independent variable. In the developed RegARIMA model, all independent variables except temperature are statistically significant. In addition, the developed model includes ARIMA(1,1,1)(0,1,1)_12_ parameters. When the R^2^, MPE, and MAPE values are examined as evaluation criteria for the RegARIMA model, R^2^ value close to 1 indicates that the model is suitable, low and negative MPE value is neutral, and MAPE value below 4% indicates high accuracy of the model. The objectives of minimizing the installation time, minimizing the investment cost, minimizing the annual cost, maximizing the carbon emission reduction, maximizing the usage time, and minimizing the risk are discussed. The limits of the model are the factors such as provincial potentials, demand quantities, and goal values. For the developed mathematical programming model, using the GAMS 23.5 optimization program, the optimum result is reached in 0.25 s using a computer with Intel(R) Core(TM) i5-10210U CPU @ 1.60 GHz 2.11 GHz processor. This integrated model is compatible with a case study problem. When the goals values ​​and deviations from the goal determined in the integrated mathematical programming model are examined, it is seen that the goals of minimization of investment cost, minimization of annual cost, maximization of carbon reduction amount, and maximization of usage time have been achieved and that the goals of minimization of installation time and minimization of risk have not been achieved. When the achieved goals are examined, as seen in Table 11, investment cost, which includes investment and land costs, provided a deviation of 1300 currency units. The annual cost, which includes maintenance and operating costs, provided a deviation of 185 currency units. The goal value of the carbon reduction amount deviated by 100 tons. The usage time target value provided a high deviation value in terms of total years. For the reason of unreachable goals, it can be stated that the determined goal values ​​are handled as very small numbers. When the site selection decisions are examined as a result of the model, the following conclusions are reached: It is recommended to install in Adana, Rize, and Bayburt provinces for biomass energy. The installation decision for biomass energy accounts for 23% of the total installation decision. Also, considering the total installed power decision, this rate is 23%. Gümüşhane and Kütahya provinces are recommended for solar energy. The installation decision for solar energy constitutes 9% of the total installation decision. Also, considering the total installed power decision, this rate is 11%. Ordu, Tunceli, Şırnak, and Yalova provinces are recommended for hydroelectric energy. The installation decision for hydroelectric energy constitutes 13% of the total installation decision. Also, considering the total installed power decision, this rate is 21%. Afyon, Şanlıurfa, and Van provinces are recommended for geothermal energy. The installation decision for geothermal energy constitutes 2% of the total installation decision. Also, considering the total installed power decision, this rate is 2%. Adana, Burdur, Çanakkale, Çorum, Erzincan, Mersin, Konya, Kütahya, Sinop, Tokat, and Bartın provinces are recommended for wind energy. The installation decision for wind energy constitutes 53% of the total installation decision. Also, considering the total installed power decision, this rate is 43%.

This study integrates different methods and examines the settlement decisions of renewable energy sources with a wide perspective. When the studies in the related literature are examined, such a large-scale study has not been found, and it is thought that this study will shed light on future studies. Moreover, in future studies, the other renewable energy sources such as hydrogen energy and wave energy can be considered. The goal programming model developed with scalarization methods can be compared. The number of used goals can be increased, or goals can be changed. Moreover, the study under consideration can be used for different locations.

## Data Availability

Not applicable.
